# Longitudinal cellular and humoral immune responses following COVID-19 BNT162b2-mRNA-based booster vaccination of craft and manual workers in Qatar

**DOI:** 10.3389/fimmu.2025.1557426

**Published:** 2025-03-27

**Authors:** Remy Thomas, Ahmed Zaqout, Bakhita Meqbel, Umar Jafar, Nishant N. Vaikath, Abdullah Aldushain, Adviti Naik, Hibah Shaath, Neyla S. Al-Akl, Abdi Adam, Houda Y. A. Moussa, Kyung C. Shin, Rowaida Z. Taha, Mohammed Abukhattab, Muna A. Almaslamani, Nehad M. Alajez, Abdelilah Arredouani, Yongsoo Park, Sara A. Abdulla, Omar M. A. El-Agnaf, Ali S. Omrani, Julie Decock

**Affiliations:** ^1^ Translational Oncology Research Center, Qatar Biomedical Research Institute (QBRI), Hamad Bin Khalifa University (HBKU), Qatar Foundation (QF), Doha, Qatar; ^2^ Communicable Disease Center, Hamad Medical Corporation (HMC), Doha, Qatar; ^3^ College of Health and Life Sciences (CHLS), Hamad Bin Khalifa University (HBKU), Qatar Foundation (QF), Doha, Qatar; ^4^ Neurological Disorders Research Center, Qatar Biomedical Research Institute (QBRI), Hamad Bin Khalifa University (HBKU), Qatar Foundation (QF), Doha, Qatar; ^5^ Diabetes Research Center, Qatar Biomedical Research Institute (QBRI), Hamad Bin Khalifa University (HBKU), Qatar Foundation (QF), Doha, Qatar; ^6^ Clinical Core, Qatar Biomedical Research Institute (QBRI), Hamad Bin Khalifa University (HBKU), Qatar Foundation (QF), Doha, Qatar; ^7^ College of Medicine, Qatar University, Doha, Qatar

**Keywords:** SARS-CoV-2, BNT162b2, booster, immune response, immunological memory

## Abstract

**Background:**

In March 2020, the rapid spread of SARS-CoV-2 prompted global vaccination campaigns to mitigate COVID-19 disease severity and mortality. The 2-dose BNT162b2-mRNA vaccine effectively reduced infection and mortality rates, however, waning vaccine effectiveness necessitated the introduction of a third vaccine dose or booster.

**Aim:**

To assess the magnitude and longevity of booster-induced immunity, we conducted a longitudinal study of SARS-CoV-2 specific cellular and humoral immune responses among Qatar’s vulnerable craft and manual worker community. We also investigated the impact of prior naturally acquired immunity on booster vaccination efficacy.

**Methods:**

Seventy healthy participants were enrolled in the study, of whom half had prior SARS-CoV-2 infection. Blood samples were collected before and after booster vaccination to evaluate immune responses through SARS-CoV-2 specific ELISpots, IgG ELISA, neutralization assays, and flow cytometric immunophenotyping.

**Results:**

T cell analysis revealed increased Th1 cytokine responses, marked by enhanced IFN-γ release, in recently infected participants, which was further enhanced by booster vaccination for up to 6-months. Furthermore, booster vaccination stimulated cytotoxic responses in infection-naïve participants, characterized by granzyme B production. Both natural SARS-CoV-2 infection and booster vaccination induced robust and durable SARS-CoV-2 specific humoral immune responses, with high neutralizing antibody levels. Prior natural infection was also linked to an increased number of class-switched B cells prior to booster vaccination.

**Conclusions:**

These findings underscore the importance of booster vaccination in enhancing anti-viral immunity across both infection-naïve and previously infected individuals, enhancing distinct arms of the anti-viral immune response and prolonging naturally acquired immunity.

## Introduction

The emergence of severe acute respiratory syndrome coronavirus 2 (SARS-CoV-2) in 2019 led to a global pandemic. SARS-CoV-2, believed to have originated from bats in Wuhan, China, rapidly spread to humans through zoonotic and human-to-human transmission (1). The virus causes Coronavirus Disease 19 (COVID-19), a highly infectious disease with a range of clinical manifestations from asymptomatic infections to severe respiratory failures. Among infected individuals, 10-15% required hospitalization, with 15-20% of those needing intensive care (2, 3). Similar to other coronaviruses, SARS-CoV-2 is a single-stranded, positive-sense RNA that encodes structural proteins that are essential for viral entry, replication and assembly including the spike (S), envelope (E), membrane (M) and nucleocapsid (N) proteins (4). The spike protein in particular plays a critical role in the virus’ pathogenesis by binding the angiotensin-converting enzyme 2 (ACE2) receptor on host cells, facilitating viral entry (5). Mutations in the spike protein can greatly impact virus transmissibility, as observed with the Alpha, Delta and Omicron variants of concern (VOCs) (6). As SARS-CoV-2 evolved, distinct genetic variants emerged, displaying altered transmission rates, disease severity and ability to escape immunosurveillance. Moreover, the duration of infectiousness evolved, with individuals infected with Omicron exhibiting an earlier onset of infectiousness compared to those infected with the Delta variant, accelerating viral transmission (7).

To reduce the global health burden of COVID-19, public health measures and global vaccination campaigns were rapidly implemented. The World Health Organization (WHO) approved 21 vaccines, including vaccines based on inactivated viruses, protein, recombinant adenovirus, DNA, and messenger RNA (mRNA) (8). Among all vaccines, the Pfizer/BioNTech (BNT162b2) and Moderna (mRNA-1273) mRNA vaccines were widely administered. While a single dose provided only limited protection (9–11), a two-dose regimen significantly improved vaccine effectiveness (VE), with Pfizer achieving > 90% effectiveness shortly after the second dose and Moderna over 80% (12–16). Nevertheless, a meta-analysis of 18 studies revealed a decline in pooled VE from 83% at one month post-vaccination to 22% at five months, with a sharp drop after 100 days following two-dose vaccination with BNT162b2 (Pfizer-BioNTech), mRNA-1273 (Moderna) and Ad26.COV2.S (Janssen) (17, 18). A large study of 10.6M individuals further demonstrated a decline in BNT162b2 VE from 94.5% at two months after the first dose to 66.6% at seven months, while mRNA-1273 VE declined from 95.9% to 80.3% over the same timespan (18). It has been postulated that the waning protection of the two-dose vaccination regimen may be attributable to both a decline in immunity and the emergence of VOCs. For instance, in a period during which the Alpha VOC was the most prevalent the two-dose VE reached 85.7% for BNT162b2 and 93.7% for mRNA-1273, whereas in a period dominated by the Delta VOC the BNT162b2 and mRNA-1273 VE values declined to 63.5% and 75.6% (19). Notably, one study investigating the effect of pre-vaccination natural immunity on two-dose BNT162b2 and mRNA-1273 VE reported that vaccine-mediated protection declines independently of prior natural infection (20). Without bias correction, they found a pooled VE of 91.3% at 14 days post second-dose which declined to 50.8% at 7 months post vaccination. Similarly, bias-corrected VE at 7 months post-vaccination reached 53.2%. In line with declining vaccine effectiveness, two doses of the BNT162b2 vaccine were shown to elicit humoral and adaptive immune responses for up to five months after the first dose (21). Analysis of six healthy, adult vaccine recipients showed an early increase in anti-spike antibody responses after the first dose (day 20), followed by a second increase after the second dose (day 34) and subsequent decline at 150 days. Anti S1-specific T cell responses mirrored this pattern, with the second dose enhancing T cell responses in all six recipients, four of whom exhibited detectable responses up to five months after the first dose. Thus, the waning of vaccine-induced immunity and emergence of VOCs prompted the need for booster vaccination to restore protection. In Qatar, BNT162b2 VE sharply dropped to below 40% at 181-270 days following the second dose, whereas administration of a third dose or booster increased VE to approximately 80% (22). Similarly, three-dose BNT162b2 and mRNA-1273 VE values at 4-11 months post second dose were comparable to two-dose VE values at 1 to 2 months post second dose in a predominantly white, non-Hispanic population (23). Notably, anti-spike antibody levels have been found to peak at 90 days after the first mRNA vaccine dose (BNT162b2, mRNA-1273), drop by day 180, and increase 2.5-fold compared to day 90 following vaccination with the third dose before gradually declining from day 251 to day 535 (24). Overall, administering a third dose of either BNT162b2 or mRNA-1273 enhanced antibody level persistence with a slower decline compared to two-dose regimens. Booster vaccination with BNT162b2 also enhanced antibody avidity, with higher levels at 6 months post-third dose compared to mRNA-1273 (25).

In this study, we examined the effects of a third dose of the BNT162b2 vaccine on cellular and humoral immune responses in craft and manual workers (CMWs) in Qatar. Previous studies highlighted that this community, comprising approximately 80% of Qatar’s population, experiences higher infection rates due to overcrowded living and working conditions and educational barriers (26–28). Given their higher vulnerability, we sought to investigate how a third vaccine dose impacts immune responses in this population and whether prior natural Sars-CoV-2 infection influences vaccine-induced immunity. Using diverse approaches, we observed that administering a third dose of the BNT162b2 vaccine effectively induced both cellular and humoral immune responses in our CMW population, while also enhancing pre-existing immunity in previously infected participants.

## Materials and methods

### Study population

A total of 70 healthy adults from the CMW community in Qatar were included in the study. All participants were enrolled in our study when they presented at the Communicable Disease Center, Qatar between May 25, 2022 and July 4, 2022 for their third BNT162b2 vaccine dose as part of the national vaccination program. We only included individuals who received two prior doses of the BNT162b2 mRNA vaccine, as verified through Qatar’s centralized electronic medical system. Demographic data and information on prior PCR-confirmed Sars-CoV-2 infection (Feb 1, 2020 onwards) were also extracted from the centralized electronic medical records (**Table 1**). As part of the Qatar national testing framework, all suspected Sars-CoV-2 infections were tested by PCR and automatically updated in the electronic medical records. None of the individuals tested positive for SARS-CoV-2 infection within 4 weeks prior to the scheduled booster dose, were immunocompromised due to underlying disease or medical treatment, or were pregnant.

**Table 1:** Study cohort demographics.

	n	(%)
Age		
18-49	64	(91)
50+	6	(9)
sex		
female	6	(9)
male	64	(91)
race		
Asian	65	(93)
White	4	(6)
African	1	(1)
nationality		
Bangladesh	9	(13)
Egypt	2	(3)
India	48	(69)
Lebanon	1	(1)
Nigeria	1	(1)
Pakistan	1	(1)
Philippines	3	(4)
Sri Lanka	4	(6)
United Kingdom	1	(1)
co-morbidities		
diabetes mellitus	1	(1)
high blood pressure	3	(4)
prior SARS-CoV-2 infection		
infection-naïve	35	(50)
earlier infection	18	(26)
recent infection	17	(24)

### Sample collection and processing

Peripheral blood was collected in 10ml EDTA blood tubes at three timepoints; at the time of the third dose (timepoint 1), 3-months after the third dose (timepoint 2) and 6-months post third dose (timepoint 3). Serum was collected after centrifugation at 3000 rpm for 10 minutes and stored at -80°C. Peripheral blood mononuclear cells (PBMCs) were isolated from freshly collected blood samples using SepMate™ density gradient centrifugation (85460; Stem Cell Technologies) according to the manufacturer’s guidelines. Next, isolated PBMCs were resuspended in freezing media (50% FBS, 40% serum-free Roswell Park Memorial Institute 1640 medium (RPMI), 10% Dimethyl sulfoxide) and stored in liquid nitrogen until further use.

### Enzyme-linked immunosorbent spot

We used two distinct ELISpot assays to quantify the number of immune cells that secrete either IFN-γ (3420-4AST-P1-1; Mabtech, Nacka Strand, Sweden) or granzyme B (3486n-4APW-P1-1; Mabtech, Nacka Strand, Sweden) in response to a pool of SARS-CoV-2 peptides. Immune cell reactivity was measured against 166 peptides derived from the S1 domain of the spike protein (amino acids 13-685, divided into two peptide pools S1 and S2) and 47 synthetic peptides, covering the spike, nucleoprotein, membrane protein, ORF3a and ORF7a (SNMO peptide pool). ELISpot assays were conducted according to the manufacturer’s guidelines using 2.5x10e5 PBMCs/well in duplicate with a final concentration of 2 ug/ml of each peptide. In addition, wells with PBMCs alone (unstimulated) were used as negative control, and PBMCs treated with an anti-human anti-CD3 antibody (mAb CD3-2, #3420-4HST-10, Mabtech, Nacka Strand, Sweden) overnight served as positive control. After 48 hours of incubation with the peptides at 37°C, PBMCs were removed, the plates were washed, and spots were developed. For each individual, the number of spot forming units (SFUs) obtained for the negative controls were subtracted from the sample values at the respective timepoints. To enable detection of secreted IFN-γ, plates were incubated with 7-B6-1-biotin detection antibody for 2 hours, followed by 1 hour incubation with Streptavidin-HRP and addition of TMB substrate. Detection of granzyme B SFUs was obtained using the MT8610-biotin detection antibody, Streptavidin-ALP and BCIP/NBT-Plus substrate according to the manufacturer’s instructions. In addition to the IFN-γ and granzyme B ELISpot assays, we also performed an IgG ELISpot assay to enumerate B cells that are secreting human IgG in response to the Sars-CoV-2 receptor binding domain (RBD) (3850-4HPW-R1-1; Mabtech, Nacka Strand, Sweden). Moreover, to gain insight into the magnitude and longevity of the humoral SARS-CoV-2 immune response of vaccinated individuals, PBMCs were pre-stimulated with R848 (1 μg/ml) and recombinant human IL-2 (10 ng/ml) for 3 days to promote the differentiation of memory B cells into antibody-secreting cells, enabling their quantification through measurement of IgG secretion. Next, pre-stimulated and unstimulated cells were seeded in duplicate at 2.5x10e5 cells/well in the ELISpot plate which was pre-coated with anti-human IgG monoclonal antibodies. After 48 hours at 37°C, RBD-specific IgG spots were detected using a WASP-tagged RBD protein, followed by anti-WASP-HRP and TMB substrate. Finally, for each ELISpot assay the number of SFUs were determined using the AID iSpot ELISpot reader (Autoimmun Diagnostika GmbH, Strasburg, Germany). Representative images are depicted in **Supplementary Figure S1**-**S3**.

### SARS-CoV-2 IgG/IgM enzyme-linked immunosorbent assay

In addition to the IgG ELISpot assay, secretion of SARS-CoV-2 specific IgG/IgM antibodies was determined using an in-house developed ELISA. In short, 96-well plates (Nunc, Maxisorp) were coated overnight at 4°C with 1μg/ml SARS-Cov-2 spike protein or Nucleoprotein in 0.2M NaHCO_3_ (pH 9.6). Plates were washed three times with PBST (0.05% Tween-20) and blocked for 1 hour at room temperature using PBST-2.25% gelatin. Next, diluted serum samples (1:800) were added to washed plates for 2 hours (room temperature, 100 rpm), followed by incubation with either goat anti-human IgG-HRP or goat anti-human IgM-HRP for 1 hour at room temperature. Finally, TMB substrate was added for 20min, and absorbance values were measured at 450nm using the EnVision^®^ Multilabel Plate Reader (PerkinElmer).

### SARS-CoV-2 neutralizing antibody assay

To assess the presence of SARS-CoV-2 antibodies with neutralizing abilities, we utilized an in-house neutralization antibody (NAb) assay. Briefly, recombinant hACE2 protein (1μg/ml in 0.2 M NaHCO_3_, pH 9.6) was coated on 96-well ELISA plates (Maxisorp, Nunc) at 4°C overnight. Plates were washed three times with PBST (0.05% Tween-20) and blocked with 2.25% gelatin in PBST for 1 hour at room temperature. Serum samples were diluted (1:10) and preincubated with 100 ng/ml RBDmFc (Genscript) in blocking buffer for 1 hour at room temperature, after which they were added to the pre-coated ELISA plate for 1 hour. In parallel, anti-SARS nanobody NbS72-Biv (500 ng/ml) was preincubated with RBDmFc (Genscript) to serve as neutralization control. Next, wells were incubated with goat anti-mouse Fc-HRP (1:10,000) for 1 hour, followed by TMB substrate for 20min. Absorbance values were measured at 450nm using the EnVision^®^ Multilabel Plate Reader (PerkinElmer).

### Immune cell phenotyping by flow cytometry

We characterized the presence of T and B cell subpopulations using DuraClone IM T cells (Beckman Coulter; #B53328) and DuraClone IM B cell tubes (#B53318, Beckman Coulter) respectively. A total of 3.0x10e5 PBMCs were resuspended in stain buffer (#554656, BD Pharmingen™) and added to the DuraClone tubes, which were vortexed for 5 seconds and incubated for 15 min in the dark at room temperature. Next, the cells were washed in DPBS (#14190-144, Gibco) and resuspended in PBS prior to analysis on the LSRFortessaTM X-20 flow cytometer (BD Biosciences) using FACS Diva Software (BD Biosciences). For each sample, 30,000 events were recorded, and further analysis was performed using FlowJo™ Software (BD Biosciences, version 10.8). Representative gating strategies are provided in **Supplementary Figure S4** and **Supplementary Figure S5**.

### Statistical analysis

Statistical analyses were performed using GraphPad PRISM V9.5.1 (GraphPad Software, CA, USA). Data normality was assessed by Shapiro-Wilk test and differences between groups were analyzed using the unpaired t-test or one-way ANOVA test with Tukey correction. A p value ≤ 0.05 was considered significant.

## Results

### Natural infection and booster vaccination differentially stimulate SARS-Cov-2 specific cellular immune responses

**Figure 1:**
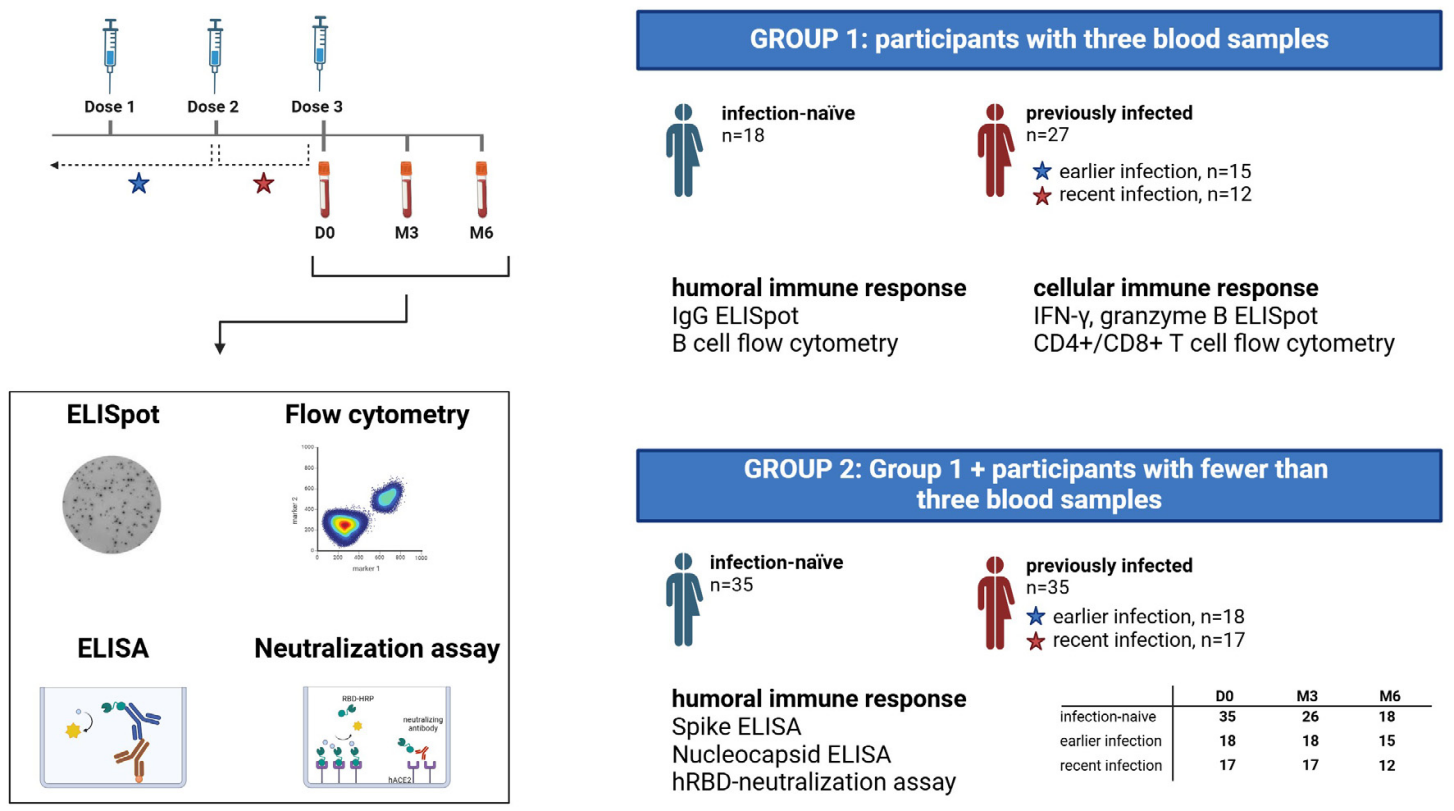
Study design diagram. Flowchart depicting the study cohort, blood sample collection and assays performed. A total of 70 participants were enrolled in the study, of whom 45 participants had blood samples collected at three timepoints (group 1), while the remaining 25 participants had fewer than 3 blood samples available (constituting group 2 together with participants from group 1). PCR-confirmed Sars-CoV-2 infection status was extracted from electronic medical records, and previously infected participants were subdivided into two groups based on the time of infection; earlier infection and recent infection. D0, Day 0; M3, 3-month post-booster; M6, 6-months post-booster.

A total of 70 participants were enrolled in the study. For 45 study participants (group 1), we collected blood samples at all three timepoints; immediately prior to the third vaccine dose (Day-0, D0), three months post-booster (Month-3, M3) and six months post-booster (Month-6, M6). Among these 45 participants, 27 had a documented SARS-CoV-2 infection before their booster dose, as verified through their electronic medical records, while 18 had no prior PCR-confirmed infection (**Figure 1**). These blood samples were used to assess cellular and humoral immune responses through ELISpot and flow cytometry analyses. In addition to the 45 participants, we included 25 participants from whom less than three blood samples were obtained (group 2) to evaluate anti-SARS-CoV-2 specific antibody levels, including the level of antibodies with neutralizing activity (**Figure 1**).

**Figure 2:**
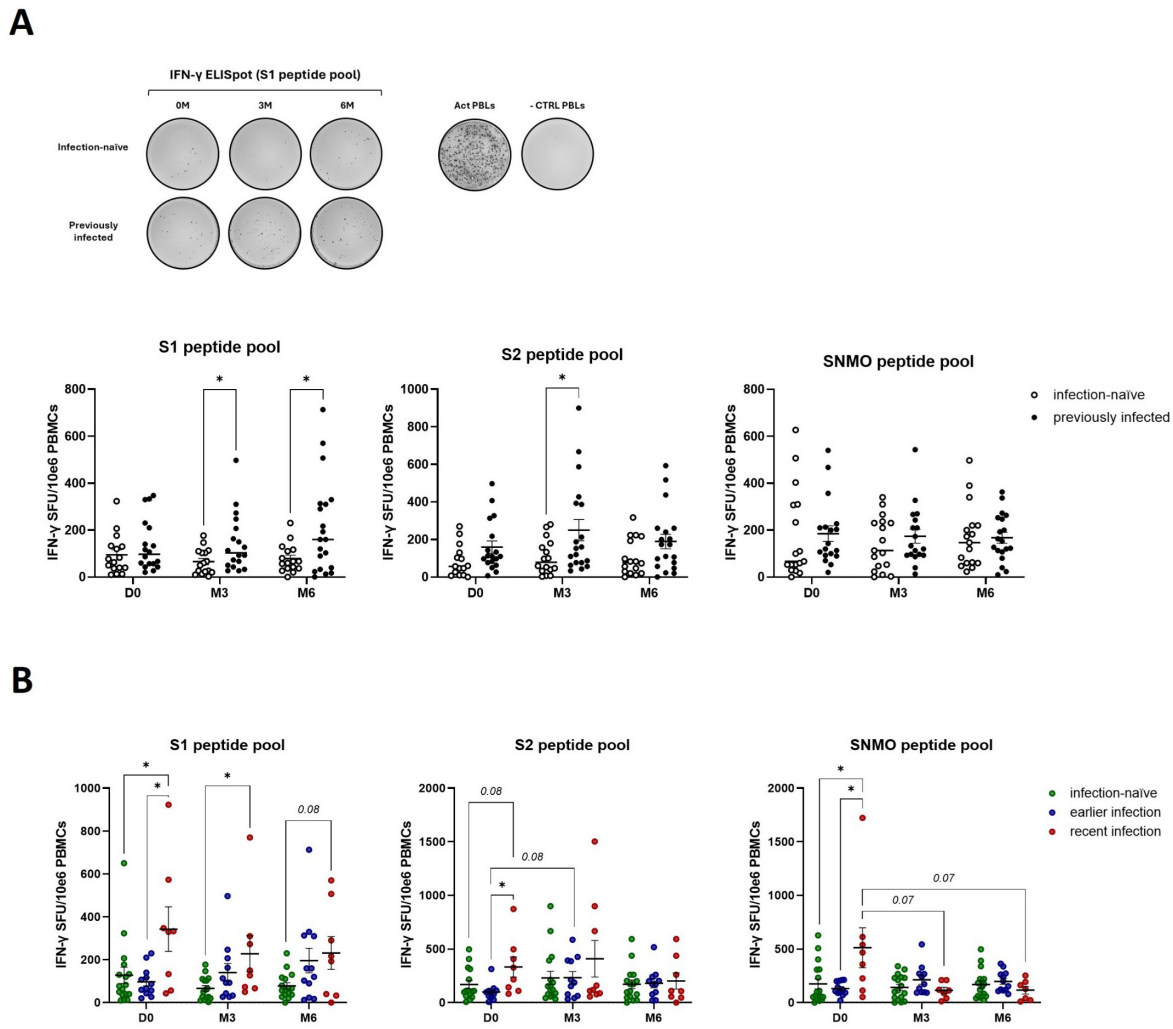
Natural exposure to SARS-CoV-2 enhances IFN-γ cellular immune responses. **(A)** Quantification of anti-spike and anti-SNMO specific IFN-γ responses in infection-naïve and previously infected participants before and after booster vaccination, as determined by IFN-γ ELISpot analysis. Representative ELISpot image for anti-S1 responses in infection-naïve and previously infected participants, with CD3-activated PBLs and unstimulated PBLs (-CTRL PBLs) as positive and negative control respectively. **(B)** Quantification of anti-spike and anti-SNMO specific IFN-γ responses in infection-naïve and previously infected participants, stratified by the time of infection. Scatter dot plots represent mean with standard error of mean (± SEM). Statistical analysis performed using unpaired Student’s t-test or one-way ANOVA with Tukey correction. *p ≤ 0.05. D0, Day 0; M3, 3-month post-booster; M6, 6-months post-booster; PBLs, peripheral blood lymphocytes; Act PBLs, activated PBLs.

Previously infected participants exhibited increased anti-spike T cell responses (S1 and S2 peptide pools) at three- and six-months post-booster (M3, M6) compared to infection-naïve participants, as measured by IFN-γ secretion of peripheral blood lymphocytes following incubation with SARS-CoV-2 peptides (**Figure 2A**). This is likely the combined result of an, albeit non-significant, elevated baseline (D0) response and the boosting effect from the third vaccine dose. No significant differences in anti-SNMO IFN-γ responses were observed in relation to previous infection or booster vaccination. Next, we stratified the previously infected participants based on the timing of infection: earlier infection (infection before second dose), and recent infection (infection between second and third dose). Upon stratification, we observed higher pre-booster anti-spike IFN-γ responses (D0 - S1 and S2) in individuals with a recent infection as compared to infection-naïve individuals or those with earlier infections (**Figure 2B**). However, booster vaccination did not significantly increase anti-spike responses within each participant group (infection-naïve, earlier infection, recent infection). In addition to elevated pre-booster anti-spike responses, recently infected participants exhibited higher baseline IFN-γ responses against the SNMO peptide pool, although these responses declined post-booster to similar levels as observed in infection-naïve and earlier infected participants. This suggests that more recent infections induce stronger anti-spike and anti-SNMO T cell responses, which naturally taper off with time and are not further enhanced or sustained by booster vaccination.

**Figure 3:**
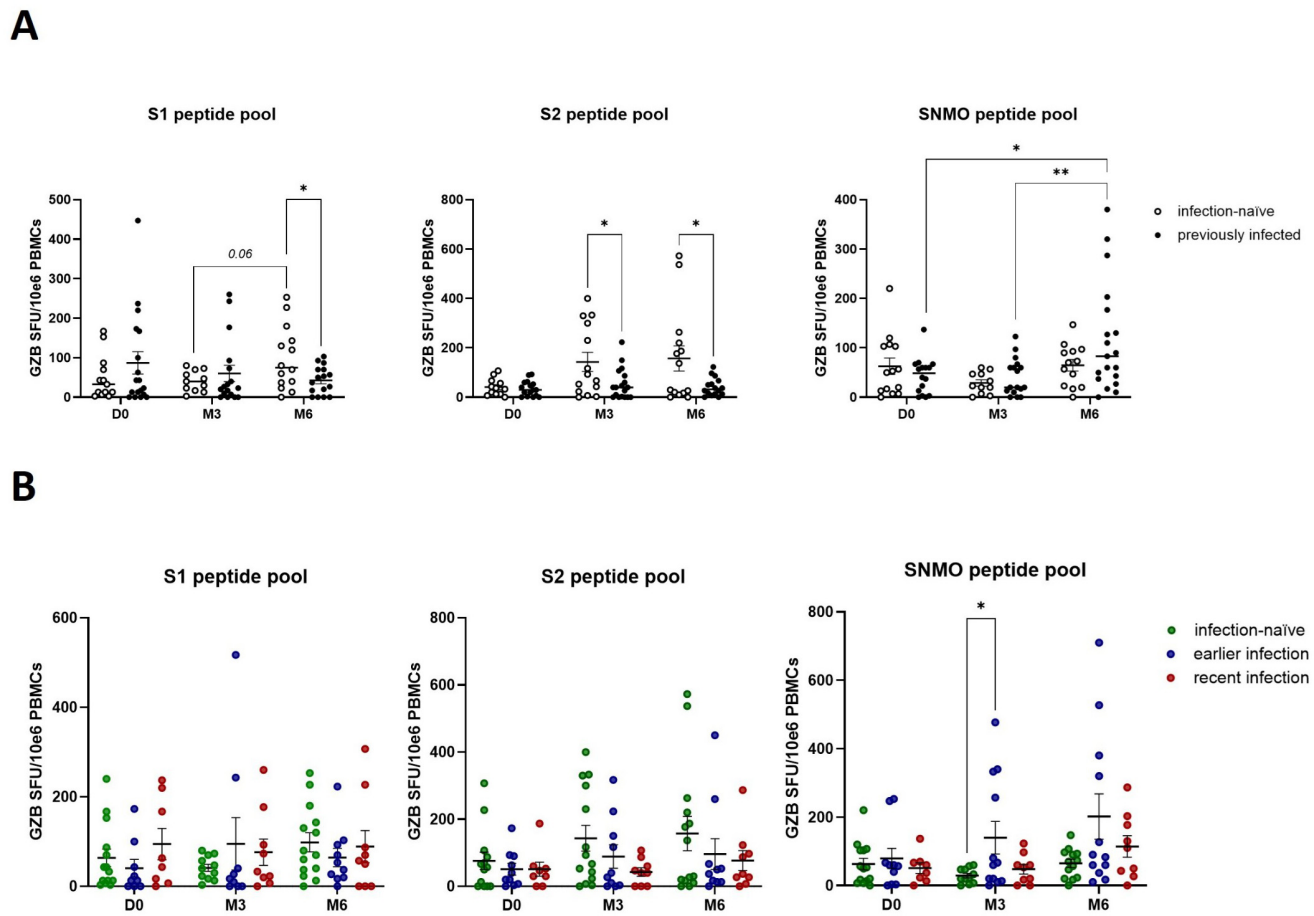
SARS-CoV-2 specific cytotoxic immune responses are enhanced by booster vaccination in the absence of prior natural infection. **(A)** Quantification of anti-spike and anti-SNMO specific granzyme B responses in infection-naïve and previously infected participants pre- and post-booster vaccination, as determined by granzyme B ELISpot analysis. **(B)** Quantification of anti-spike and anti-SNMO specific granzyme B responses in infection-naïve and previously infected participants, stratified by the time of infection. Scatter dot plots represent mean with standard error of mean (± SEM). Statistical analysis performed using unpaired Student’s t-test or one-way ANOVA with Tukey correction. *p ≤ 0.05, **p ≤ 0.01. D0, Day 0; M3, 3-month post-booster; M6, 6-months post-booster.

To assess the effect of booster vaccination and pre-booster Sars-CoV-2 infection on cytotoxic cellular responses, we measured granzyme B release by peripheral blood lymphocytes in response to SARS-CoV-2 peptide pools. Pre-booster (D0), no significant differences in the number of granzyme B-producing cells were observed between infection-naïve and previously infected participants (**Figure 3A, B**). Following booster vaccination, we found a borderline significant (p=0.06) increase in anti-spike granzyme B responses, in particular anti-S1, in infection-naïve participants (S1 – M3 versus M6) (**Figure 3A**). Furthermore, booster vaccination enhanced late anti-SNMO responses in previously infected participants (SNMO – M6 versus D0 and M3). No differences were found when previously infected participants were stratified by the time of infection, except for a 3-months post-booster significant increase in SNMO-responses in earlier infected participants compared to infection-naïve participants (SNMO – M3) (**Figure 3B**). These findings suggest that natural SARS-CoV-2 infection primarily primes memory Th1 cytokine responses, characterized by IFN-γ release, whereas booster vaccination induces granzyme B-mediated cytotoxic responses, which primarily involve CD8+ T cell and NK cell activity, particularly in infection-naïve participants.

### Natural infection induces robust SARS-CoV-2 specific humoral responses with neutralizing abilities, which can be enhanced by booster vaccination

**Figure 4:**
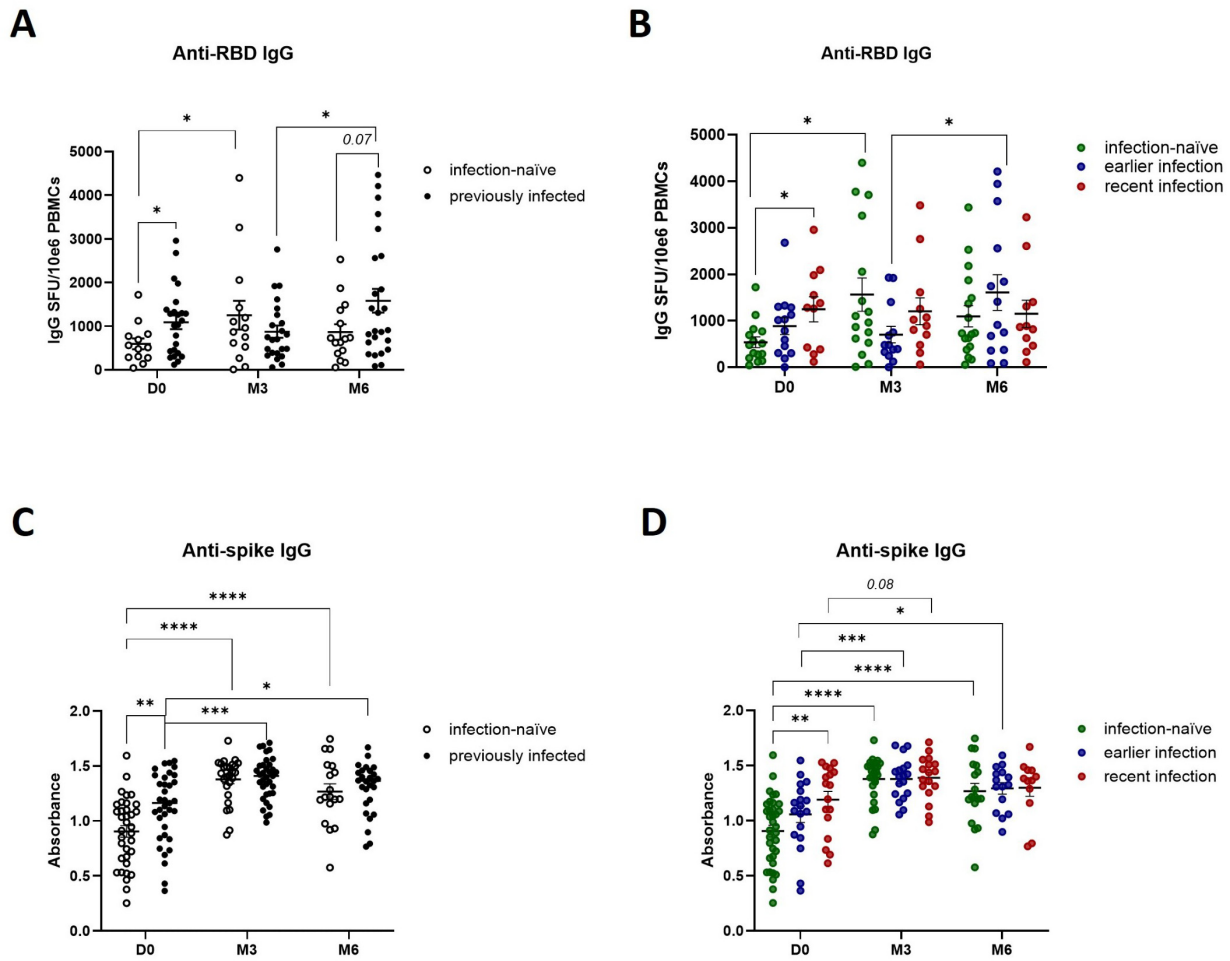
Effect of prior natural infection and booster vaccination on anti-SARS-CoV-2 RBD and spike IgG levels. **(A)** Quantification of anti-RBD IgG producing memory B cells in infection-naïve and previously infected participants before and after booster vaccination, as determined by anti-RBD IgG ELISpot analysis. **(B)** Quantification of anti-RBD IgG producing memory B cells in infection-naïve and previously infected participants, stratified by the time of infection. **(C)** Quantification of anti-spike IgG levels in infection-naive and previously infected participants, as determined by in-house ELISA of pre- and post-booster samples. **(D)** Quantification of anti-spike IgG levels in infection-naive and previously infected participants, stratified by time of infection. Scatter dot plots represent mean with standard error of mean (± SEM). Statistical analysis performed using unpaired Student’s t-test or one-way ANOVA with Tukey correction. *p ≤ 0.05, **p ≤ 0.01, ***p ≤ 0.001, ****p ≤ 0.0001. D0, Day 0; M3, 3-month post-booster; M6, 6-months post-booster.

**Figure 5:**
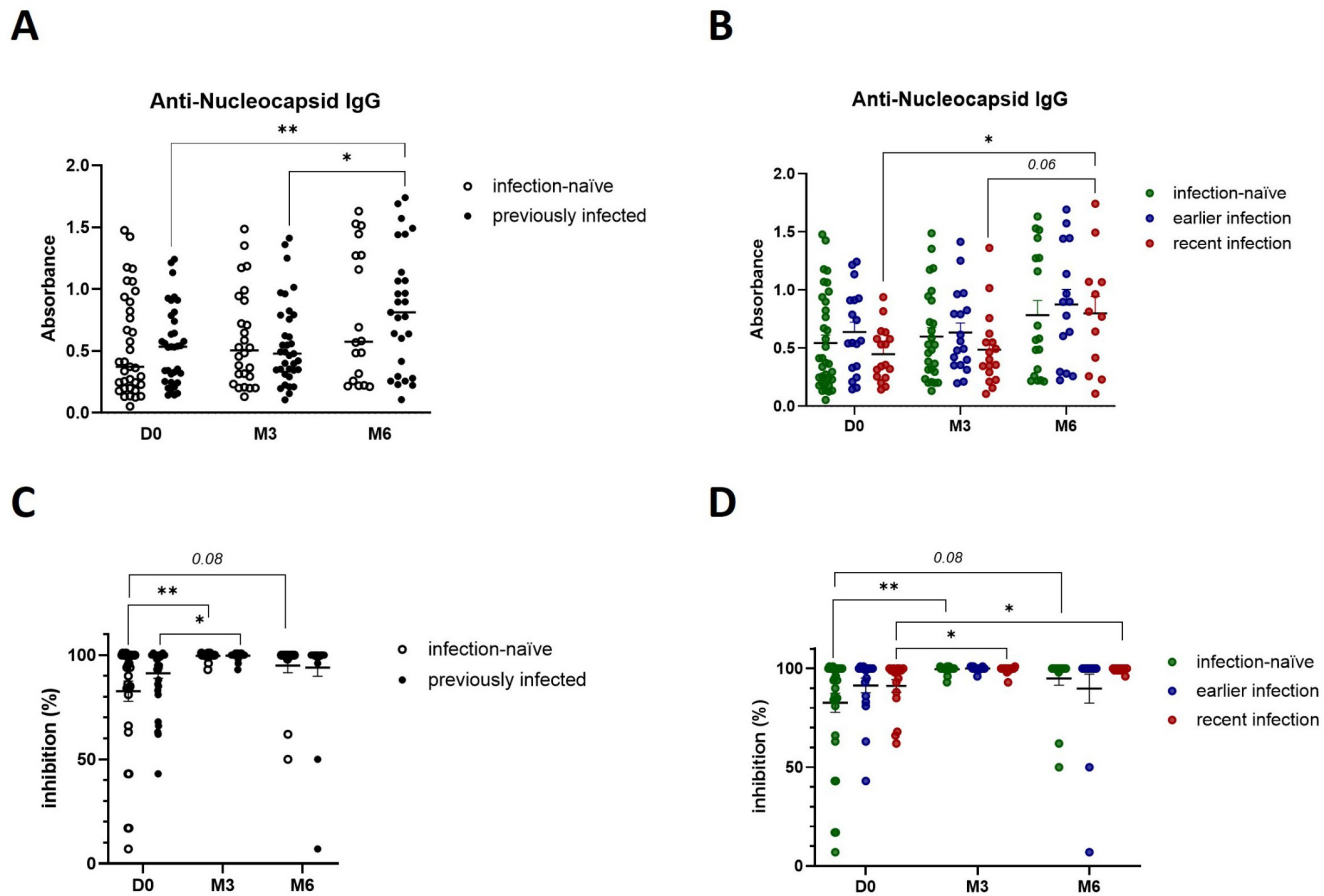
Booster vaccination increases the presence of SARS-CoV-2 neutralizing antibodies. **(A)** Anti-nucleocapsid IgG levels in infection-naïve and previously infected participants pre- and post-booster vaccination, as determined by in-house ELISA. **(B)** Anti-nucleocapsid IgG production in infection-naïve and infected participants, stratified by the time of infection. **(C)** Quantification of SARS-CoV-2 IgG with neutralizing ability in infection-naïve and previously infected participants pre- and post-booster vaccination, as determined by in-house neutralization assay. **(D)** Analysis of SARS-CoV-2 neutralizing IgG antibodies in infection-naïve and infected participants, stratified by the time of infection. Scatter dot plots represent mean with standard error of mean (± SEM). Statistical analysis performed using unpaired Student’s t-test or one-way ANOVA with Tukey correction. *p ≤ 0.05, **p ≤ 0.01. D0, Day 0; M3, 3-month post-booster; M6, 6-months post-booster.

Based on our observations that SARS-CoV-2 natural infection and booster vaccination likely prime different components of the antiviral cellular immune response – specifically Th1 cytokine and granzyme B-mediated cytotoxic responses - we next investigated their impact on SARS-CoV-2 specific humoral immune responses. Prior to booster vaccination, previously infected participants displayed a higher number of IgG-secreting memory B cells (**Figure 4A**), particularly those with recent infections (**Figure 4B**). Furthermore, booster vaccination induced a transient increase in IgG-positive memory B cells in infection-naïve participants, with a peak at 3-months before declining at 6-months, while in participants with earlier infections the number of IgG-positive memory B cells peaked at 6-months (**Figure 4A, B**). This suggests that previously infected participants display a robust and durable humoral memory response which can be activated upon antigen re-exposure, while booster vaccination can further enhance B cell responses. To confirm these findings, we developed an ELISA to detect IgGs against the full-length spike protein. In accordance with our IgG ELISpot results, previously infected individuals, particularly those with recent infections, exhibited higher baseline IgG-secreting memory B cell responses (**Figure 4C, D**). Furthermore, we confirmed that booster vaccination enhanced those responses across all participants, including infection-naïve participants. Looking at anti-nucleocapsid IgG levels specifically, we observed a post-booster steady increase in previously infected participants, particularly those with recent infections (**Figure 5A, B**), further indicating that both natural infection and booster vaccination contribute to memory B cell responses. To further assess the functional abilities of these humoral responses, we used an in-house neutralization assay that demonstrated a post-booster increase in neutralizing antibody activity in infection-naïve and previously infected participants, particularly in recently infected participants, underscoring the role of booster vaccination in enhancing functional memory B cell responses (**Figure 5C-D**).

### Phenotyping analysis of cellular and humoral immune responses

**Figure 6:**
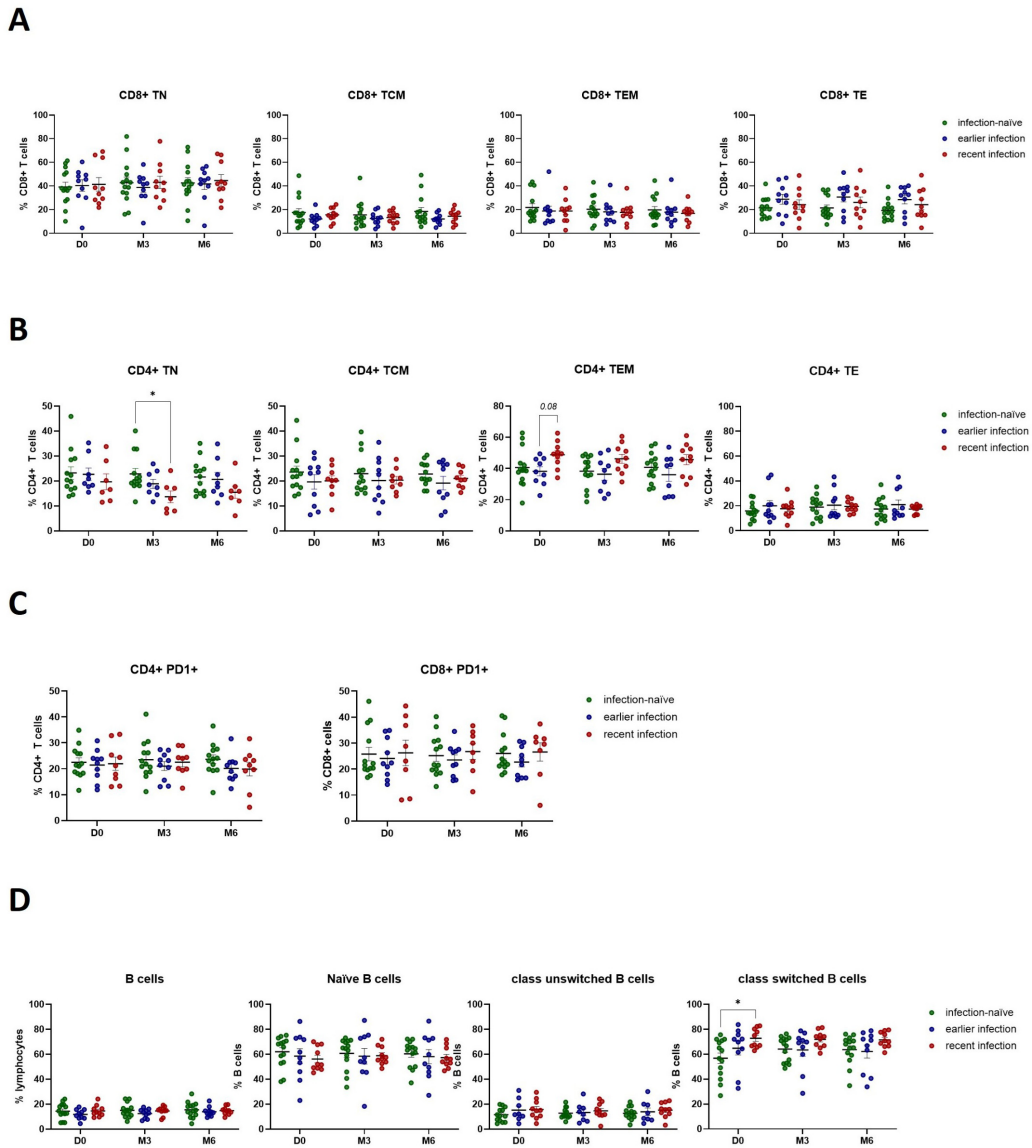
SARS-CoV-2 infection activates the anti-viral immune response by promoting CD4+ T effector memory and inducing class-switched B cell responses. **(A)** Flow cytometry analysis of CD8+ T cell phenotypes. **(B)** Flow cytometry analysis of CD4+ T cell phenotypes. **(C)** Flow cytometry analysis of PD1 expression on CD4+ and CD8+ T cells. **(D)** Flow cytometry analysis of B cell phenotypes. Scatter dot plots represent mean with standard error of mean (± SEM). Statistical analysis performed using unpaired Student’s t-test or one-way ANOVA with Tukey correction. *p ≤ 0.05. TN, naïve; TCM, central memory; TEM, effector memory; TE, effector T cells. D0, Day 0; M3, 3-month post-booster; M6, 6-months post-booster.

Given the presence of a cytotoxic cellular and neutralizing humoral immune response in participants with previous natural infection and following booster vaccination, we further investigated the immune cell phenotypes that may contribute to this immunological memory. We did not observe any differences in CD8+ T cell phenotypes that may complement the increased cytotoxic activity following natural infection and booster vaccination (**Figure 6A**). In line with our observations demonstrating the presence of an enhanced Th1 cytokine response in previously infected participants, we observed a decrease in the number of naïve CD4+ T cells (TN) following booster vaccination of recently infected participants (**Figure 6B**). In addition, they exhibited a trend (p=0.08) towards a higher number of CD4+ effector memory cells (TEM) at baseline compared to those with earlier infections. Of note, we did not find any significant changes in the number of CD4+ or CD8+ T cells expressing PD-1, suggesting that neither CD4+ nor CD8+ T cells exhibited an exhausted phenotype through PD-1/PD-L1 signaling (**Figure 6C**). Moreover, we observed a higher pre-booster number of class-switched B cells in recently infected individuals, which was sustained for up to 6 months post-booster (**Figure 6D**), underscoring the impact of natural infection in the development of a robust and durable memory B cell response which can be re-activated in response to booster vaccination.

## Discussion

**Figure 7:**
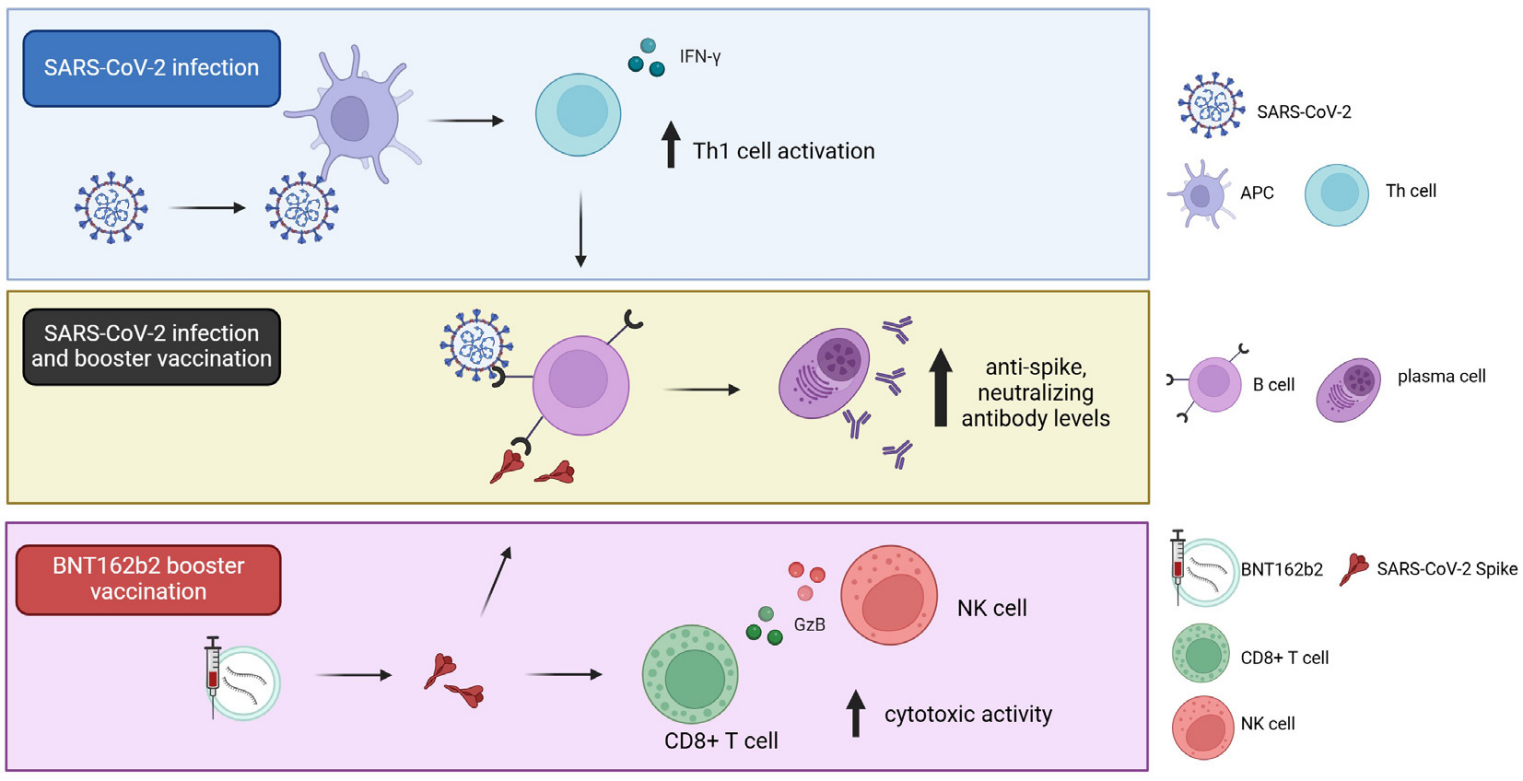
Diagram depicting the effect of SARS-CoV-2 natural infection and BNT162b2 third dose vaccination on cellular and humoral immune responses. Prior natural infection induces Th1 cytokine and humoral immune responses. Booster vaccination with BNT162b2 further enhances humoral immune responses, and promotes a cytotoxic cellular response in infection-naïve individuals.

In March 2020, the WHO declared COVID-19 a pandemic, making it the first pandemic caused by a coronavirus. Given its high infection and mortality rate, global efforts focused on limiting the spread of the SARS-CoV-2 virus through implementation of nation-wide vaccination campaigns. According to data from Our World in Data (updated August 14^th^, 2024), 64.8% of the global population (approximately 5.18 billion people) has completed the initial COVID-19 vaccination protocol, which consists out of two doses for most vaccines (29). Following the success of the initial vaccine approaches, vaccine effectiveness waned over time, raising the question whether a booster dose could mitigate the decline in protection. Worldwide, 54% of individuals who completed the initial vaccination protocol (approximately 2.82 billion people) also received a booster dose (29). Here, we present a comprehensive longitudinal analysis of cellular and humoral immune responses against SARS-CoV-2 pre- and post-booster vaccination and provide insights into the role of prior natural infection in establishing immunological memory (**Figure 7**).

In our study, we demonstrate that previously infected participants exhibit robust humoral immune responses, characterized by higher pre-booster anti-spike antibody levels, including those of neutralizing antibodies. Those responses were further enhanced by booster vaccination. In addition, the recently infected participants showed an increased number of class-switched B cells before receiving the third vaccine dose, indicative of a durable B cell memory response. In infection-naïve participants, booster vaccination resulted in a sustained increase in the number of anti-spike neutralizing antibodies, reaching levels comparable to those observed in previously infected individuals. These findings are in line with a Danish study that reported prolonged anti-spike antibody persistence following vaccination with a third vaccine dose of BNT162b2 or mRNA-1273 (24). Furthermore, although previously infected participants showed a stronger baseline anti-spike humoral response than infection-naïve participants, we found similar responses following the booster vaccination, corroborating the previous study by Andrejko et al. that reported comparable vaccine effectiveness at 7-months post-vaccination, regardless of pre-vaccination naturally acquired immunity (20).

To further characterize the anti-viral immune response, we assessed any changes in the cellular immune response and found that different arms of the cellular immune response were preferentially induced by either naturally acquired or vaccination-induced immunity. Recent exposure to SARS-CoV-2 primarily induced a durable Th1 cytokine response with increased IFN-γ production, and a non-significant trend towards an increased number of CD4+ T effector memory cells. In contrast, booster vaccination more readily induced granzyme-B mediated cytotoxic immune responses, predominantly involving both T and NK cell activity, in infection-naïve participants for up to 6-months post-booster. We did not find any increase in the number of PD-1 positive CD4+ or CD8+ T cells, suggesting a lack of PD-1 mediated T cell exhaustion up to 6-months following the third vaccine dose. This raises the question whether cellular immune responses could be sustained for more than 6-months post-booster. Future studies should further assess the magnitude, longevity and exhaustion status of immunological memory beyond 6-months post-booster. Moreover, it would be of interest to study additional immune checkpoint markers and specific exhaustion markers (TOX, TCF1 and CXCL13) across CD4+ and CD8+ T cell subpopulations including T effector memory cells and tissue-resident memory T cells. Collectively, our findings indicate that prior natural SARS-CoV-2 infection and booster vaccination both stimulate the humoral immune response while activating different aspects of the cellular immune response, and that these responses can persist for at least 6-months after receiving the third vaccine dose of BNT162b2.

In conclusion, we demonstrate that booster vaccination plays a critical role in enhancing the anti-viral humoral and cellular immune responses regardless of infection history, likely providing broader protection in the population. However, it remains to be determined whether booster-induced immune responses and/or naturally acquired immunity confer protection against emerging Sars-CoV-2 variants. A recent epidemiological study identified two distinct protection patterns against reinfection based on VOC dominance. While pre-Omicron infections provided strong and durable protection against reinfection, infections during the time when Omicron was dominant more effectively prevented reinfection within the first 3 to 6 months post-infection (30). These recent findings highlight the importance of continuous monitoring of Sars-CoV-2 viral spread and evolution to better inform vaccination strategies.

## Data Availability

The original contributions presented in the study are included in the article/supplementary material. Further inquiries can be directed to the corresponding author.
