# Supplementary figures and images for: Longitudinal cellular and humoral immune responses following COVID-19 BNT162b2-mRNA-based booster vaccination of craft and manual workers in Qatar
Source: Front Immunol. 2025 Mar 27;16:1557426. doi: 10.3389/fimmu.2025.1557426 (PMC11983602; doi:10.3389/fimmu.2025.1557426)

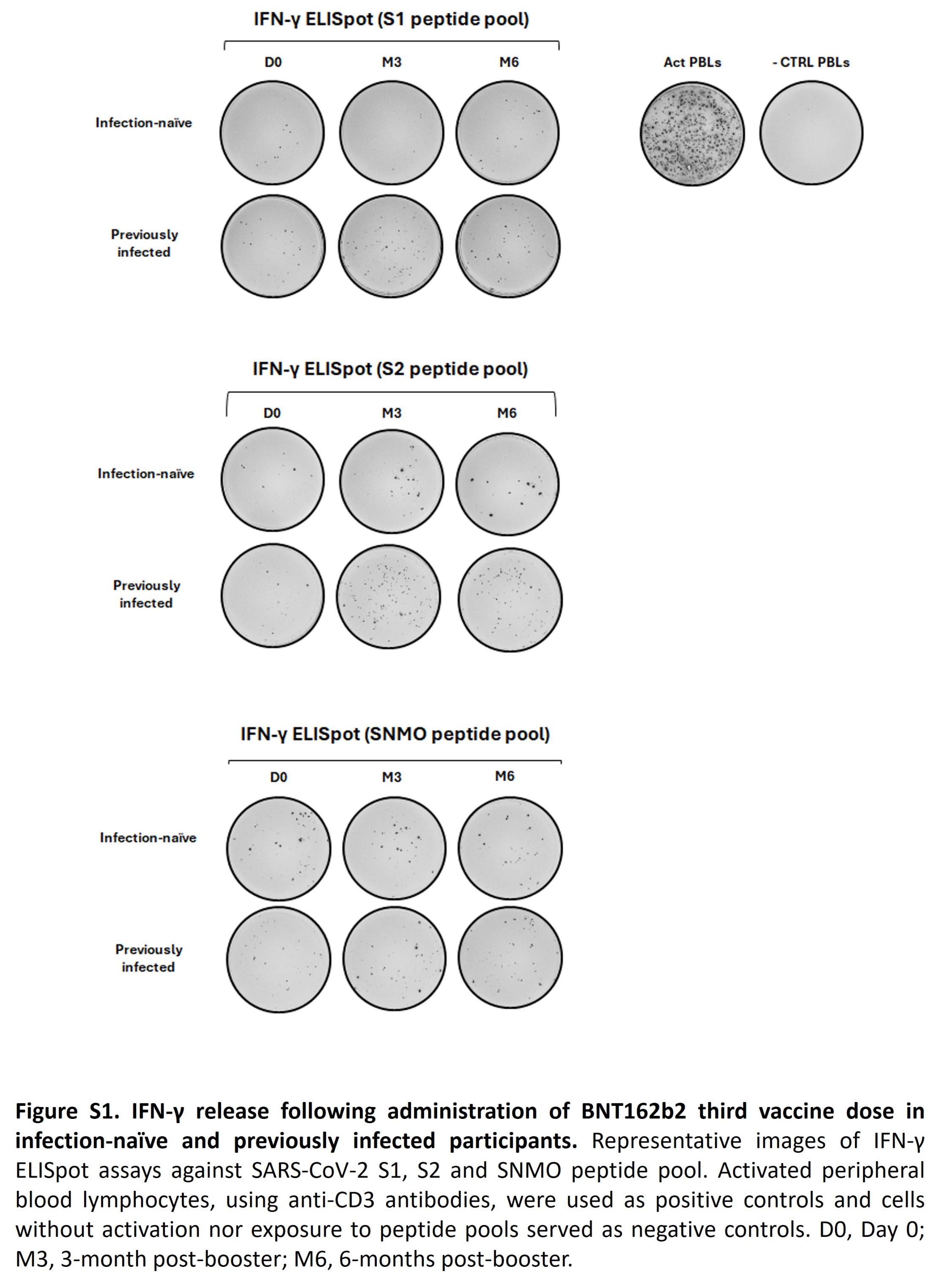

Supplement: Supplementary file 1 [file Image1.jpeg]

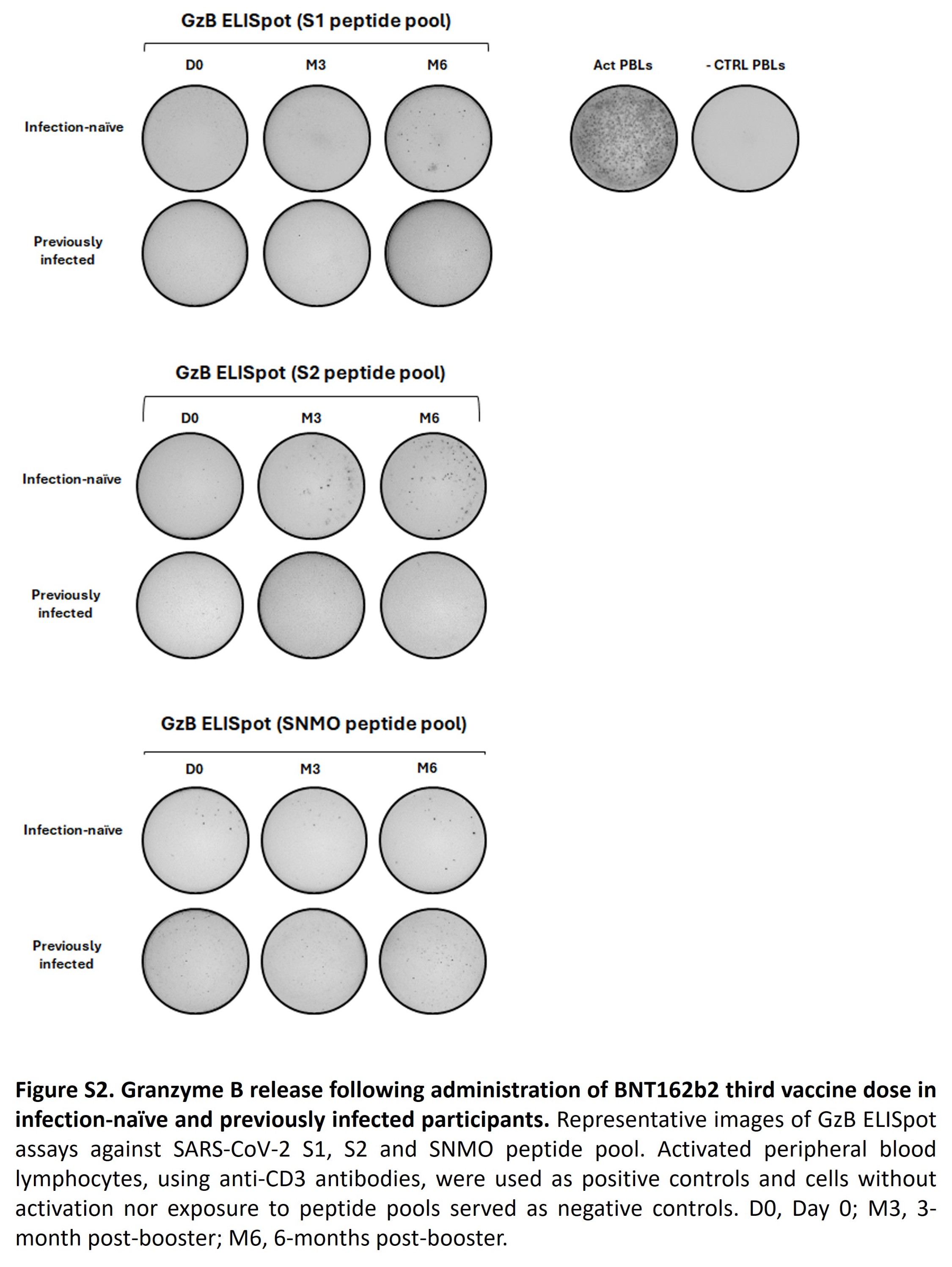

Supplement: Supplementary file 2 [file Image2.jpeg]

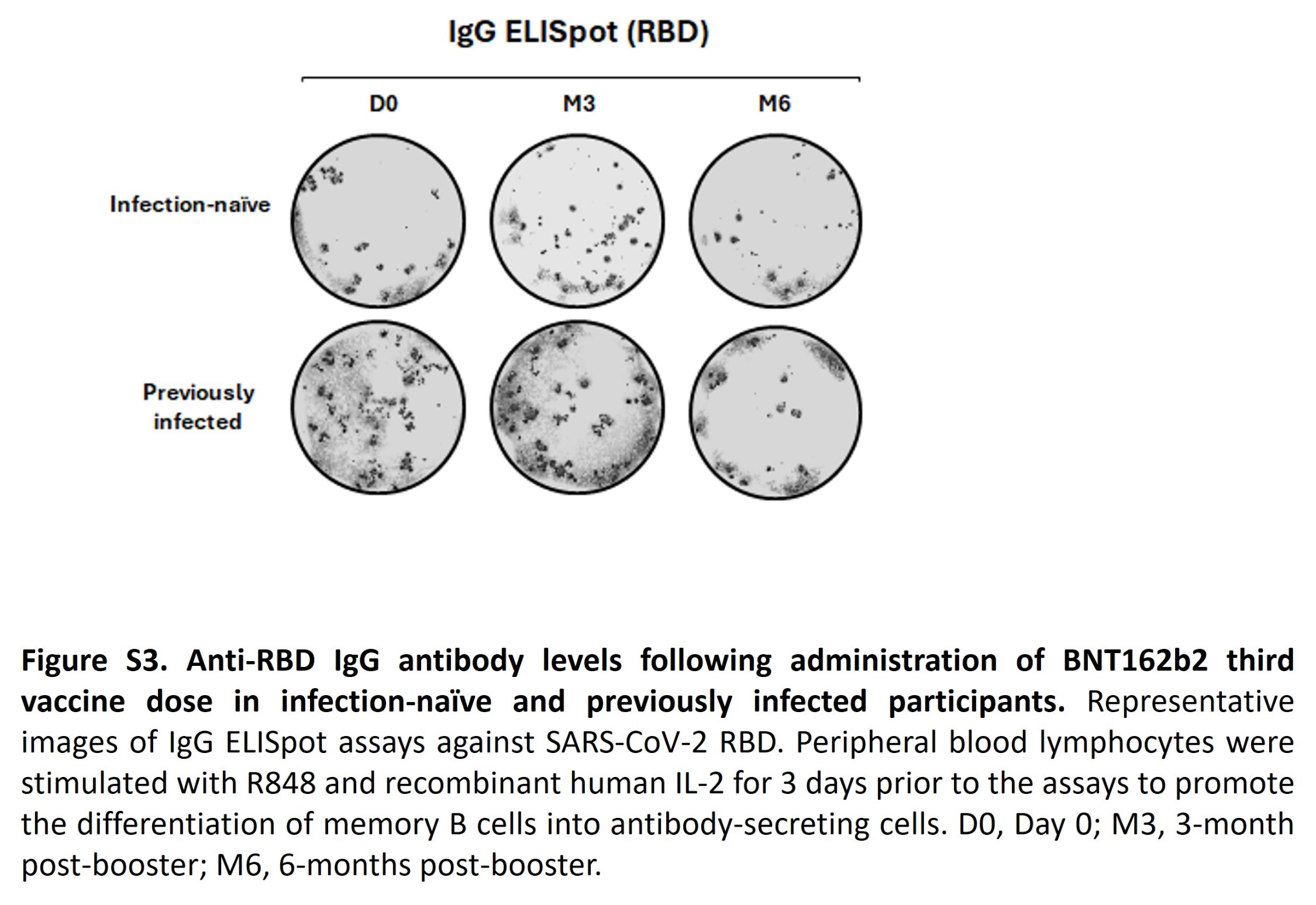

Supplement: Supplementary file 3 [file Image3.jpeg]

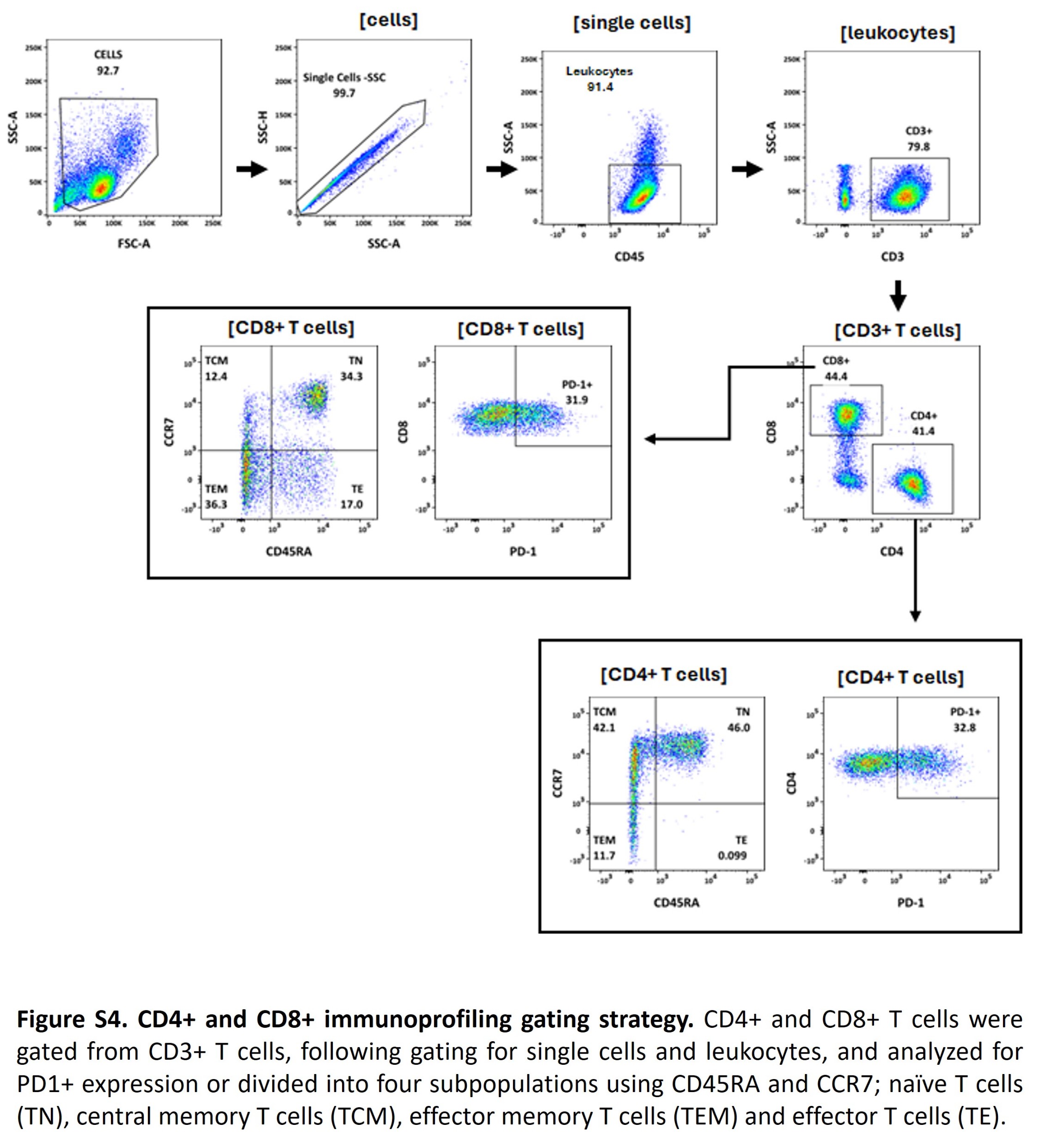

Supplement: Supplementary file 4 [file Image4.jpeg]

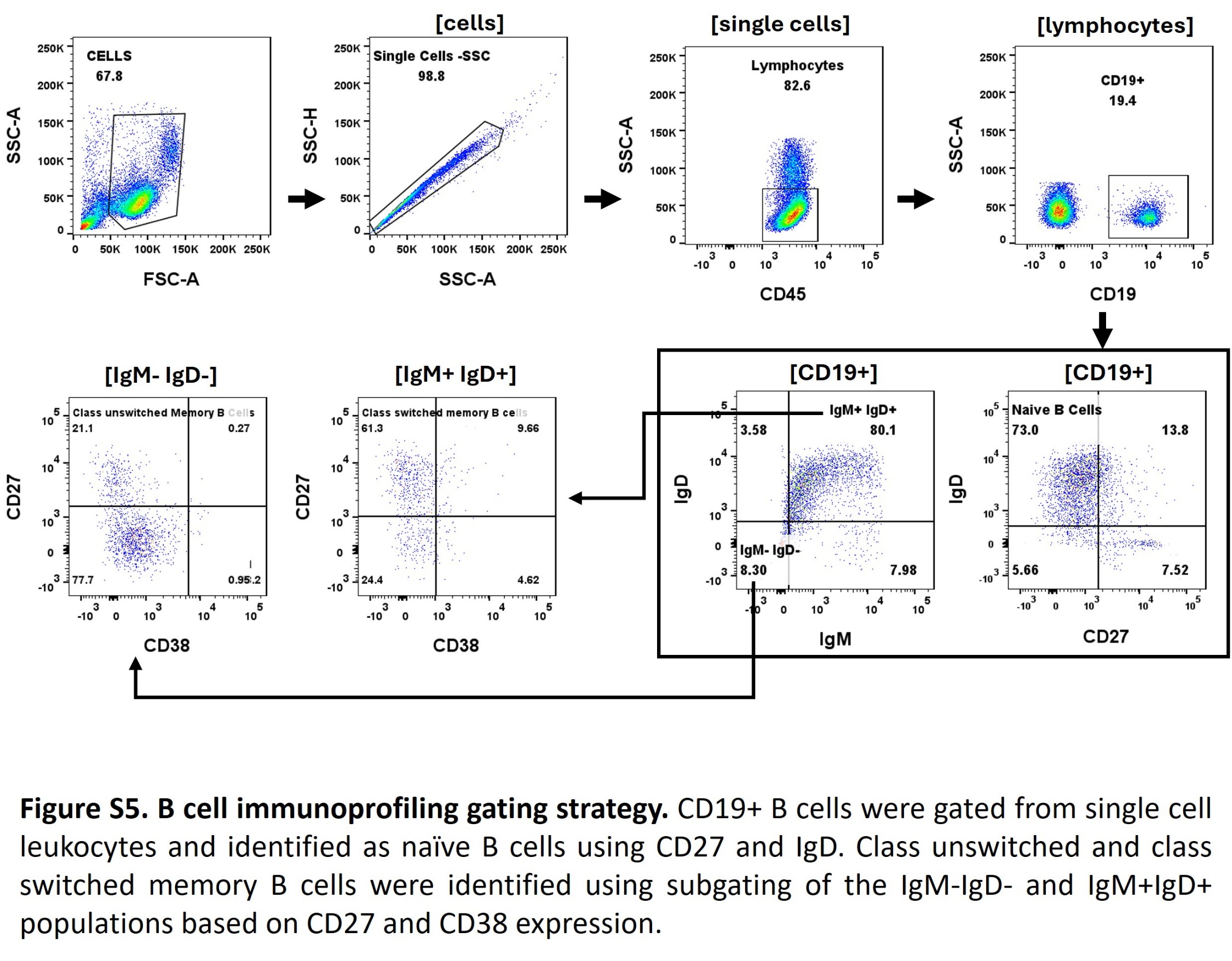

Supplement: Supplementary file 5 [file Image5.jpeg]
